# Multiscale Poincaré plots for visualizing the structure of heartbeat time series

**DOI:** 10.1186/s12911-016-0252-0

**Published:** 2016-02-09

**Authors:** Teresa S. Henriques, Sara Mariani, Anton Burykin, Filipa Rodrigues, Tiago F. Silva, Ary L. Goldberger

**Affiliations:** 1 Wyss Institute for Biologically Inspired Engineering at Harvard University, Boston, MA USA; 2 Center for Anesthesia Research and Excellence (CARE), Beth Israel Deaconess Medical Center, Boston, MA USA; 3 Margret and H.A. Rey Institute of Nonlinear Dynamics in Physiology and Medicine, Division of Interdisciplinary Medicine and Biotechnology, Beth Israel Deaconess Medical Center, Boston, MA USA; 4 Division of Sleep and Circadian Disorders, Department of Medicine, Brigham and Women’s Hospital, Harvard Medical School, Boston, MA USA; 5 Department of Physics, Faculty of Sciences, University of Lisbon, Lisbon, Portugal

**Keywords:** Atrial fibrillation, Complexity, Congestive heart failure, Fractal, Heart rate, Multiscale, Nonlinear dynamics, Poincaré plot, Time series, Visualization

## Abstract

**Background:**

Poincaré delay maps are widely used in the analysis of cardiac interbeat interval (RR) dynamics. To facilitate visualization of the structure of these time series, we introduce *multiscale Poincaré* (MSP) plots.

**Methods:**

Starting with the original RR time series, the method employs a coarse-graining procedure to create a family of time series, each of which represents the system’s dynamics in a different time scale. Next, the Poincaré plots are constructed for the original and the coarse-grained time series. Finally, as an optional adjunct, color can be added to each point to represent its normalized frequency.

**Results:**

We illustrate the MSP method on simulated Gaussian white and 1/f noise time series. The MSP plots of 1/f noise time series reveal relative conservation of the phase space area over multiple time scales, while those of white noise show a marked reduction in area. We also show how MSP plots can be used to illustrate the loss of complexity when heartbeat time series from healthy subjects are compared with those from patients with chronic (congestive) heart failure syndrome or with atrial fibrillation.

**Conclusions:**

This generalized multiscale approach to Poincaré plots may be useful in visualizing other types of time series.

## Background

The use of delay (also called return) maps is central to the qualitative and quantitative analysis of dynamical systems [1, 2]. The phase space realization with dimension of two and delay of one is referred to as a Poincaré plot [1–3]. This graphical method is widely used to visualize and quantify short- and longer-term properties of heart rate variability (HRV) [3–11].

Here we propose a multiscale generalization of the Poincaré plot method, prompted by the observation that physiologic systems generate fluctuations over a broad range of scales. These fluctuations are a marker of the complexity of biologic dynamics, especially in healthy organisms under “free-running” conditions [12–15]. A variety of computational tools, including fractal and multifractal methods [16–18], multiscale entropy [19–22], and multiscale time irreversibility [23, 24] have been proposed to probe the temporal richness of physiologic signals and of their dynamical alterations with senescence and pathology.

We sought to develop a complementary graphical method to aid in visualizing the multiscale properties of cardiac interbeat interval and other types of time series, in conjunction with these computational analyses. We were further motivated by the pedagogic need for graphical techniques to assist students and trainees in developing an intuitive sense for concepts and terms such as *multiscale*, *self-similarity* (*fractality*) and *complexity loss*. To this end, we adapted and extended the methodology of delay (Poincaré) maps. Classical Poincaré maps are single-scale, since they graph the value of one data point of the original time series against the next. The novelty of our method consists in the generation of *multiscale Poincaré* (MSP) plots. This multiscale implementation is accomplished via a simple coarse-graining procedure [19, 21] that produces multiple rescaled “copies” of the original signal. For each coarse-grained time series, we create a Poincaré plot, which is then assembled into the final montage. Furthermore, as a potentially useful, but optional adjunct, the data points in each plot are color-coded using an estimated normalized probability density function to further enhance visualization of time series properties.

To introduce and illustrate the MSP method, we first apply it to synthetic Gaussian white and 1/f-type noise time series. The technique is then applied to RR interval time series obtained in health, chronic (congestive) heart failure and atrial fibrillation. The primary goal here is to introduce this method as a simple-to-implement visualization tool.

## Methods

The MSP technique consists of three steps: i) construction of the coarse-grained time series; ii) construction of a Poincaré plot for the original and each of the coarse-grained time series, and iii) colorization of the Poincaré plots based on an estimated normalized probability density function.

### Coarse-graining technique and construction of MSP montage

Considering a time series *X* of length N, *X* = {*x*_1_, *x*_2_, *x*_3_, …, *x*_*N* − 1_, *x*_*N*_}, its Poincaré plot is the scatter plot representing the set of points: (*x*_1_, *x*_2_), (*x*_2_, *x*_3_), …, (*x*_*N* − 1_, *x*_*N*_) [4–6, 8].

The coarse-grained time series [19, 21] are obtained using a non-overlapping moving average low-pass filter. The window length, s, determines the scale of the coarse-grained time series {Σ_s_(j)}. The elements of the coarse-grained time series for scale *s* are determined according to the equation:

$$ {\Sigma}_s(j)=\frac{1}{s}\sum_{i=\left(j-1\right)s+1}^{js}{x}_i,\kern2em 1\le j\le \frac{N}{s} $$

Here, the Poincaré plots for the original and the coarse-grained time series are constructed and assembled into the MSP montage.

### Colorization of MSP plots

**Fig. 1:**
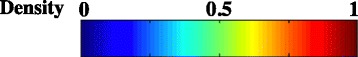
Color scheme map. The interval [0,1] is divided into 256 adjacent intervals, each of which is assigned a color following the Matlab® “jet” color scheme

The traditional monochromatic Poincaré plot can be enhanced by adding color to each of its data points to convey information about their normalized frequency of occurrence [25–27]. The probability density function can be estimated by employing the histogram technique (used here), or employing kernel density-based or other methods [27–29]. Specifically, we used the Matlab®, *dscatter* function to compute the smoothed normalized two-dimensional histogram of {(x_i_, x_i+1_)} [30]. We employed the Matlab® “jet” color-map (Fig. 1). We note that alternative color schemes [31, 32] and functions can be used for the same purpose.

## Results and discussion

For illustrative purposes, we applied the MSP method to synthetic white and 1/f noise time series and to RR intervals time series in health and selected pathologic states.

### MSP plots for synthetic white and 1/f noise time series

**Fig. 2:**
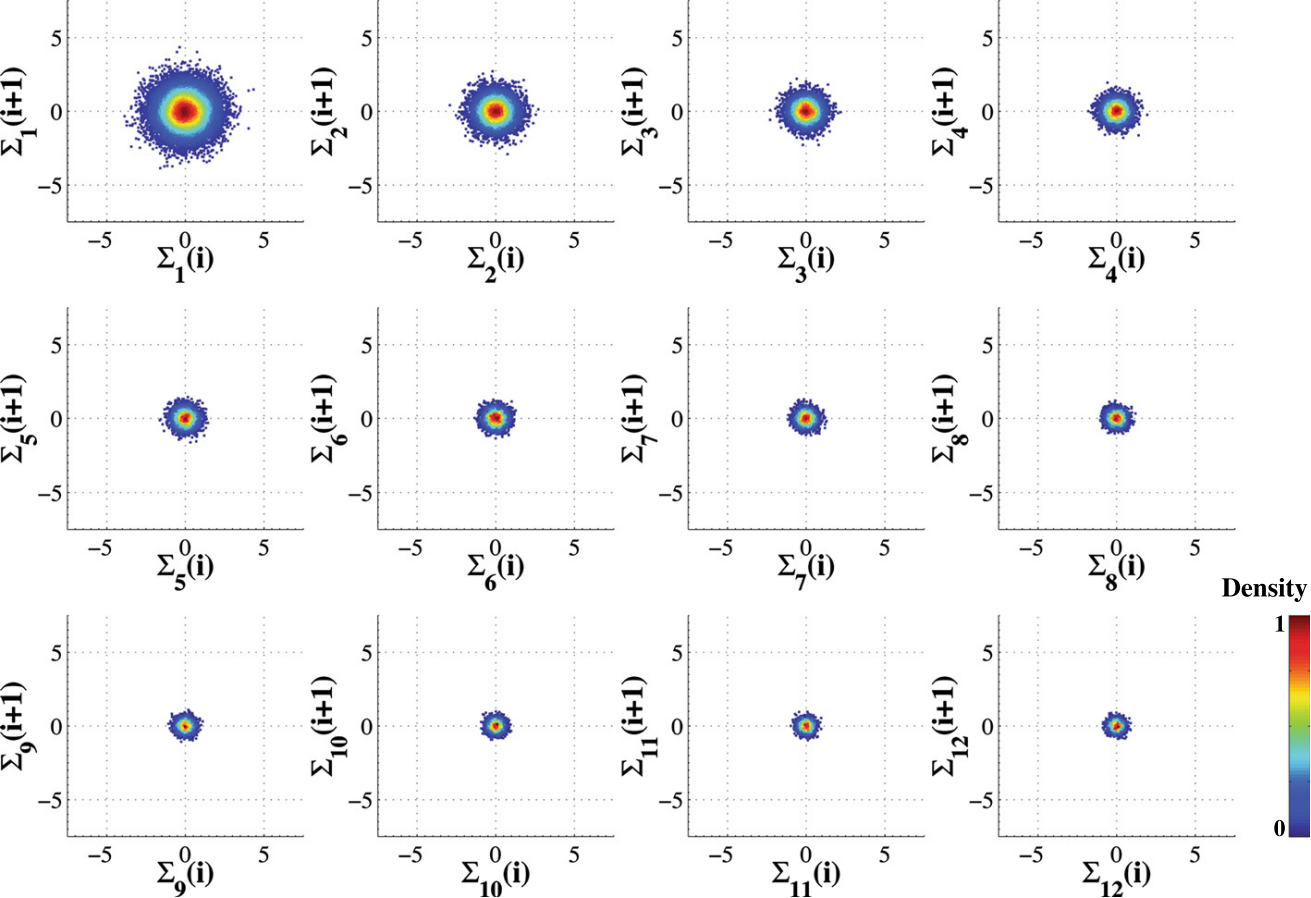
Multiscale Poincaré (MSP) plots of synthetic white noise time series (20,000 data points) for scales *s* = 1 to 12. Note that **Σ**_***s***_ (i) represents the i^th^ data point of the coarse-grained time series for scale s. The data points are colorized based on their estimated normalized probability density function (see text and Fig. 1)

Figure 2 shows the MSP montage for a Gaussian white noise time series comprising 20,000 data points. The traditional Poincaré plot (equivalent to scale 1) has a circular shape, due to the normal distribution of the uncorrelated data points (where a uniformly distributed random time series would be represented by a square shape). As expected, the Poincaré plots for scales >1 show the same mean (centroid) with a progressive decrease in circular area. This decrease is due to the relation between the radius of these plots and the standard deviation of each time series. The coarse-graining procedure, by averaging consecutive uncorrelated random points, creates time series with consecutively lower variance. Specifically, the variance of each coarse-grained time series decreases with the scale as σ_s_^2^ = σ^2^ /s, where s is the scale and σ^2^ and σ_s_^2^ represent the variance of the original and coarse-grained time series, respectively.

**Fig. 3:**
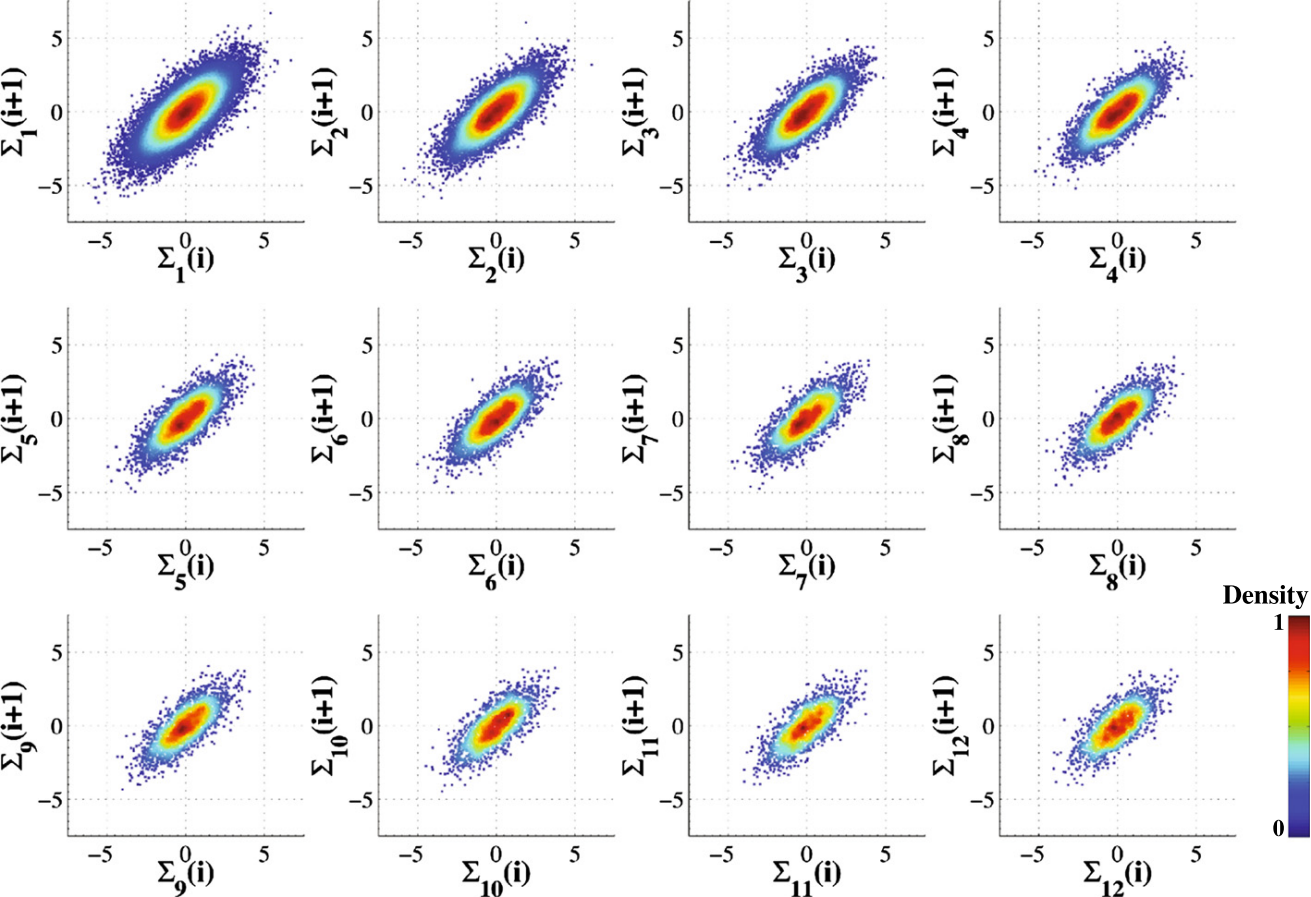
Multiscale Poincaré (MSP) plots of synthetic 1/f noise time series (20,000 data points) for scales *s* = 1 to 12. Note that **Σ**_***s***_ (i) represents the i^th^ data point of the coarse-grained time series for scale *s*. The data points are colorized based on their estimated normalized probability density function (see text and Fig. 1)

Figure 3 shows the MSP montage for a 1/f time series, which represents a complex, fractal structure characterized by correlations between data points across multiple time scales. The conventional (single-scale) Poincaré plot of a 1/f noise time series has an elliptical shape indicating positive correlations between consecutive data points (large values more likely to be followed by large values and low values more likely to be followed by low values). The standard deviation of 1/f noise coarse-grained time series remains constant across scales [21]. Thus, the area of the Poincaré plots remains approximately constant across scales, a consequence of the fractal structure of the 1/f noise signal. (The slight decrease in the area is attributable to the filtering of very high frequency components in a finite time series).

### MSP plots for RR interval time series

The MSP technique was then applied to recordings from an open-access dataset of deidentified cardiac interbeat interval time series from Holter monitor (~24 h) recordings (http://www.physionet.org/challenge/chaos/) [33]. This database includes RR interval time series from ostensibly healthy subjects, as well as patients with congestive (chronic) heart failure (CHF) syndrome, and patients with permanent atrial fibrillation (AF). Here we describe the geometry of the MSP plots from one subject in each of these three groups, representing the extremes of health and heart disease. The MSP plots for the other subjects in each group showed similar characteristics.

#### Healthy heartbeat dynamics

**Fig. 4:**
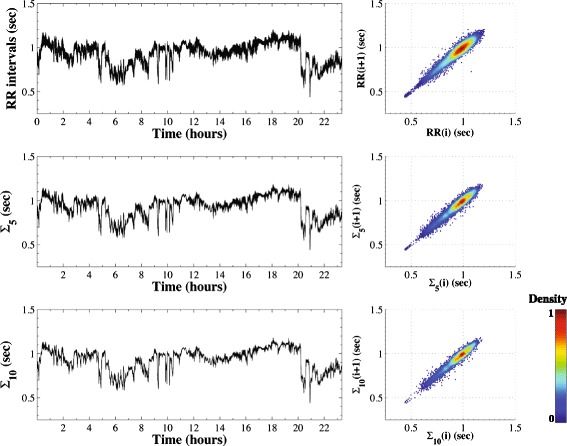
The left panels present the cardiac interbeat (RR) interval time series (top) from a healthy subject and the coarse-grained time series for scales 5 (middle) and 10 (bottom). The right panels present the corresponding Poincaré plots, which have been colorized based on their estimated normalized probability density functions (see text and Fig. 1). The original RR time series was filtered to remove outliers (using http://physionet.org/tutorials/hrv-toolkit/HRV.src/filt.c, with visual assistance). (The MSP plots are derived from dataset # n2nn from the PhysioNet database described in the text)

Figure 4 presents the RR interval time series of a healthy subject, their coarse-grained time series for scales 5 and 10 and corresponding colorized Poincaré plots. The area of these plots is maintained across scales, reminiscent of what is seen with simulated 1/f time series (Fig. 3).

**Fig. 5:**
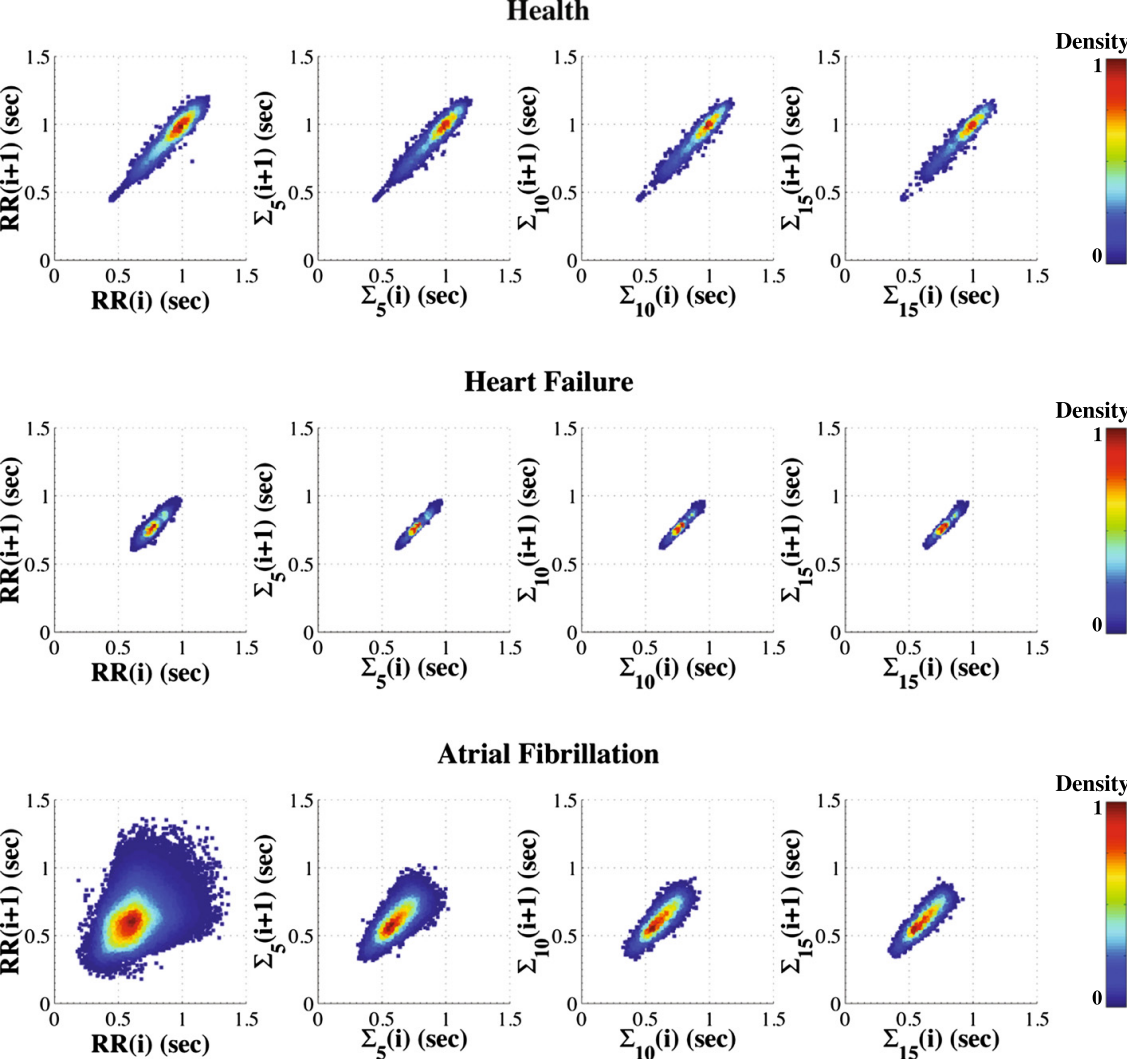
“Collapse of complexity” with severe pathology. The MSP plots are derived from a healthy subject in sinus rhythm (top), a patient with chronic heart failure (CHF) in sinus rhythm (middle) and a patient with atrial fibrillation (AF) (bottom). The MSP plots correspond to the original time series (left column) and their derived coarse-grained time series for scales 5 (second column), 10 (third column) and 15 (right column). The “wedge” shaped appearance of the AF plots at lower scales relates to the constraints on physiologic conduction imposed by refractoriness of the AV node. (The sinus rhythm time series of the healthy subject and the patient with CHF syndrome were filtered to remove outliers using http://physionet.org/tutorials/hrv-toolkit/HRV.src/filt.c, and visual assistance). We note that the scales correspond to slightly different mean rates in each subject. For example, scale 10 corresponds to means of 10 points. Thus, if the RR mean is around ~1 s (healthy case) this coarse-grained time scale will be ~10 s, but when the mean RR is around 0.6 s (AF case), the same time scale will correspond to ~6 s. (The MSP plots are from subjects # n2nn, c3nn and a5nn, respectively, from the PhysioNet database available at http://www.physionet.org/challenge/chaos/) [33]

The geometry of the traditional Poincaré plot of heartbeat intervals in health and disease has been the subject of extensive study [4, 6, 11, 34, 35]. The traditional (scale 1) Poincaré plot for healthy subjects exhibits a “comet-like” shape [4]. We confirm this tapered (teardrop) morphology [36, 37], and also find that both the overall shape of the map and its area are preserved across scales. Furthermore, the teardrop appearance for scale 1 is consistent with the previously reported correlation of the average value of the RR interval with the variance of the time series, i.e., shorter RR intervals are associated with lower dispersion (variance) of the RR intervals [4, 38]. The MSP representation (Fig. 5 - top panels) highlights information by showing that this asymmetric “tail,” present at scale 1, is preserved across scales for the healthy subject.

#### Chronic Heart Failure (CHF) and Atrial Fibrillation (AF) Dynamics

Previous reports [3–5] have shown that the area of the Poincaré plot of RR interval time series is markedly smaller for patients with severe CHF (but still in sinus rhythm) than healthy subjects. Here, we extend this finding by showing that the area is invariant under the coarse-graining operation. Our results (Fig. 5 - middle panels) are consistent with an overall reduction in multiscale complex variability with heart failure [17, 21].

Prior studies [25, 39] have shown that the Poincaré plots (scale 1) of RR interval time series from subjects with AF are reminiscent of those derived from white noise signals. MSP plots (Fig. 5 - bottom panels) highlight these results. In addition, they show that the resemblance between the Poincaré plots of the AF subject and those of white noise signals is most apparent for relatively short time scales (in this example, scales < 10, approximately lower than 15 s). In both white noise and AF cases, the areas of the Poincaré plots decrease with scale. Such behavior is attributable to the uncorrelated structure of the time series fluctuations. However, for larger time scales, the Poincaré plots for the subject with AF show the classical elliptical shape indicative of long-range correlations [40]. This finding is consistent with previous studies [40, 41] reporting that the absolute value of the scaling exponents derived from log-log power spectral plots of RR intervals time series from subjects with AF are closer to 1 (fractal noise) than to 0.5 (white noise) or 2 (brown noise) across the lower frequency bands, with a “cross-over” toward those of white noise at higher frequencies.

The persistence of correlated behavior at higher scales (lower frequencies) in AF may be related to the degree to which the atrioventricular (AV) junction and autonomic nervous system function are preserved in this common arrhythmia. Whether AF associated with the most severe derangements of AV nodal conduction (and concomitant myocardial disease) shows a complete breakdown of correlations is an intriguing question with basic and clinical implications. The MSP method may be of use in screening “big datasets” in order to gain some intuition about the multiscale behavior of RR intervals in AF in different clinical subsets. We hypothesize that permanent AF associated with heart failure would have a less complex structure by this method than so-called “lone” AF, which is not associated with clinically apparent heart disease.

**Fig. 6:**
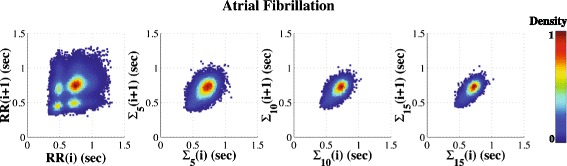
Another example of atrial fibrillation (AF). The MSP plots correspond to the original time series (left column) and their coarse-grained time series for scales 5 (second column), 10 (third column) and 15 (right column), respectively. This case of AF differs from that in Fig. 5 (bottom panels) by showing 4 sub-areas of RR clusters evident at scale 1, three of which disappear at higher scales (i.e., lower frequencies). Furthermore, the MSP plots at the higher scales are more circular than those in Fig. 5 (bottom panels), suggesting more random behavior in the case shown here at these scales. The differences between these recordings, which are not readily apparent from the original time series or other conventional representations, support the potential utility of MSP plots in exploring the dynamics of different subsets of AF. (The MSP plots are from subject # a1nn from http://www.physionet.org/challenge/chaos/) [33]

In this regard, Fig. 6 shows an example of AF [25] from another patient. The multiscale Poincaré plots reveal a different pattern of variability on both shorter and longer time scales compared with that shown in the bottom panel of Fig. 5. First is a short time scale clustering of RR intervals, embedded in the overall map. These additional clusters correspond to alternation of RR intervals which has been noted before in some cases of AF [25, 39, 42], but remains to be mechanistically explained and clinically investigated further. One possible explanation is dual-pathway AV conduction [25, 39]; another is a Wenckebach variant of conduction block in the AV node. The finding was not due to ventricular ectopy here. Second, the MSP plots add information by revealing that this anomalous pattern is *scale-specific*, as it is not apparent with coarse graining. In contrast, MSP analysis of RR time series from healthy subjects shows that the “tail” at the lower left portion of the plots (due to decreased variance with increased heart rate) is present *across scales* (Fig. 4). While “anecdotal,” these examples support the possible utility of using MSP plots in evaluating subsets of RR time series in health and disease having quantitatively and qualitatively different interbeat interval dynamics that may not be readily discernible using conventional time series inspection and analysis. More generally, the above findings are consistent with the concept that perturbations related to advanced aging and pathology (e.g., heart failure, atrial fibrillation, etc.) may be most evident in disturbances in higher frequency fluctuations, those required for “fine-tuning” adaptiveness [13, 14, 43].

#### Use of colorization

Finally, we note that the colorization of the MSP plots, an optional feature of the multiscale renderings, is intended to facilitate rapid assessment of the values of the most frequently observed pairs of RR intervals (mode) as well as of the shape of the probability density function. For example, Fig. 5 shows that the most frequently observed values are ~1 s for the healthy subject, ~0.75 s for the patient with CHF and ~ 0.5 s for the patient with AF. In addition, Fig. 5 also shows that the probability density function is skewed to the left in the case of the healthy subject and to the right in case of the CHF patient and the subject with AF on short time scales. Whether quantitative analyses developed for traditional Poincaré plots [5, 6, 8, 11, 35, 44] can be usefully extended to MSP plots is of interest but outside the scope of this brief methodological note. We also emphasize that these plots are intended to complement current quantitative methods of time series analysis (e.g., Fourier, fractal/multifractal, and entropy-related analyses, to name but a few).

## Conclusions

We introduce a novel delay map implementation termed *multiscale Poincaré* (MSP) plots, to facilitate visualization of multiscale structure of cardiac interbeat interval time series. The method comprises: i) a coarse-graining operation that generates a family of time series; ii) delay map construction for the original and the coarse-grained time series; and iii) colorization of the delay maps based on an estimated normalized probability density function. The method appears to be useful in depicting concepts such as scaling behavior in health and disease and contrasting “real-world” and simulated data. Future studies are needed to evaluate its use in heart rate dynamics, as well as its potential utility in studying other types of time series.

## Abbreviations

AF: Atrial fibrillation
AV: Atrioventricular
CHF: Congestive (chronic) heart failure
HRV: Heart rate variability
MSP: Multiscale Poincaré
